# Genomic exploration of coral-associated bacteria: identifying probiotic candidates to increase coral bleaching resilience in *Galaxea fascicularis*

**DOI:** 10.1186/s40168-023-01622-x

**Published:** 2023-08-19

**Authors:** Talisa Doering, Kshitij Tandon, Sanjida H. Topa, Sacha J. Pidot, Linda L. Blackall, Madeleine J. H. van Oppen

**Affiliations:** 1 School of BioSciences, The University of Melbourne, Parkville, VIC Australia; 2 Department of Microbiology and Immunology at the Peter Doherty Institute for Infection and Immunity, The University of Melbourne, Parkville, VIC Australia; 3 Australian Institute of Marine Science, Townsville, QLD Australia

**Keywords:** *Galaxea fascicularis*, Reactive oxygen species, Reactive nitrogen species, Nitric oxide, Oxidative stress, Coral probiotics, Bacterial genomes, Bacterial carbon translocation, semiSWEET

## Abstract

**Background:**

Reef-building corals are acutely threatened by ocean warming, calling for active interventions to reduce coral bleaching and mortality. Corals associate with a wide diversity of bacteria which can influence coral health, but knowledge of specific functions that may be beneficial for corals under thermal stress is scant. Under the oxidative stress theory of coral bleaching, bacteria that scavenge reactive oxygen (ROS) or nitrogen species (RNS) are expected to enhance coral thermal resilience. Further, bacterial carbon export might substitute the carbon supply from algal photosymbionts, enhance thermal resilience and facilitate bleaching recovery. To identify probiotic bacterial candidates, we sequenced the genomes of 82 pure-cultured bacteria that were isolated from the emerging coral model *Galaxea fascicularis*.

**Results:**

Genomic analyses showed bacterial isolates were affiliated with 37 genera. Isolates such as *Ruegeria*, *Muricauda* and *Roseovarius* were found to encode genes for the synthesis of the antioxidants mannitol, glutathione, dimethylsulfide, dimethylsulfoniopropionate, zeaxanthin and/or β-carotene. Genes involved in RNS-scavenging were found in many *G. fascicularis*-associated bacteria, which represents a novel finding for several genera (including *Pseudophaeobacter*). Transporters that are suggested to export carbon (semiSWEET) were detected in seven isolates, including *Pseudovibrio* and *Roseibium*. Further, a range of bacterial strains, including strains of *Roseibium* and *Roseovarius*, revealed genomic features that may enhance colonisation and association of bacteria with the coral host, such as secretion systems and eukaryote-like repeat proteins.

**Conclusions:**

Our work provides an in-depth genomic analysis of the functional potential of *G. fascicularis*-associated bacteria and identifies novel combinations of traits that may enhance the coral’s ability to withstand coral bleaching. Identifying and characterising bacteria that are beneficial for corals is critical for the development of effective probiotics that boost coral climate resilience.

Video Abstract

**Supplementary Information:**

The online version contains supplementary material available at 10.1186/s40168-023-01622-x.

## Introduction

Reef-building corals and coral reefs are under imminent threat from climate change. Increasing sea surface temperatures in combination with high irradiance levels, which often occur during summer heat waves driven by climate change, are the major cause of mass coral bleaching [1]. Coral bleaching is the breakdown of the obligate symbiosis between the coral host and its algal symbionts of the family Symbiodiniaceae, which results in the separation of the symbionts from the coral host tissues. This leaves the host in a carbon-deprived state [2], which is often followed by coral death and the degradation of coral reefs. There is a growing concern that ocean warming is progressing too rapidly for natural adaptation of corals to keep pace due to their relatively long generation times. This notion has led to a new field of research aimed at accelerating evolutionary processes to enhance coral bleaching resilience [3]. The concept of assisted evolution of corals includes, among other approaches, the manipulation of coral-associated microbial symbionts, such as bacteria. Coral-associated bacteria are important players in coral health and fitness as they defend the coral host from pathogens through the synthesis of antimicrobial compounds [4], produce antioxidants and cycle nutrients such as nitrogen, sulphur, carbon and phosphorus [5]. Correlations between the composition of coral-associated bacterial communities and coral heat tolerance suggest bacteria play beneficial roles in coral heat tolerance [6], but the underlying mechanisms are currently unknown. Microbiome manipulation has been successfully applied in fields like agriculture and medicine [7], but it is still in its infancy in cnidarians. Nevertheless, the feasibility of coral microbiome manipulation has recently been demonstrated [8, 9]. Further, recent success has been achieved in treating coral white pox disease [10] and stony coral tissue loss disease [11]. Studies aimed at increasing coral bleaching resilience via microbiome manipulation have also shown positive results, even though it remains to be explored if and how the added bacteria were driving the improved tolerance to heat stress. These studies inoculated corals with bacteria isolated from corals and seawater and that were tested for potentially beneficial functions (antimicrobial activities against pathogens, activity of the antioxidant enzyme catalase and the presence of genes responsible for sulphur and nitrogen cycling) and demonstrated reduced thermal bleaching and reduced phenotypic responses to pathogen infection in *Pocillopora damicornis* [12] and enhanced bleaching recovery in *Mussismilia hispida* [13]. Another study showed increased bleaching tolerance in heat sensitive *Pocillopora* sp. and *Porites* sp. that were inoculated with whole microbiomes from heat-tolerant conspecifics [14].

The main theory of bleaching is the oxidative stress hypothesis which poses that increased temperature and light damage photosystem II, reductive pentose phosphate cycle reactions and thylakoid membranes [15, 16] in the photosymbionts, which leads to an overproduction of toxic reactive oxygen species (ROS) [17]. This can overwhelm antioxidant responses and excess ROS diffuse into the coral host cells where they cause damage to macromolecules (e.g. DNA) and trigger a cellular cascade that leads to bleaching [17, 18]. Various ROS such as singlet oxygen (^1^O_2_), superoxide (O_2_^−^), hydrogen peroxide (H_2_O_2_) and hydroxyl radicals (OH^−^) are continuously produced during photosynthesis in Symbiodiniaceae even under non-stress conditions [19] and are promptly scavenged by the antioxidant defence system consisting of various enzymatic and non-enzymatic mechanisms in the photosymbiont and host cells. Scavenging enzymes include catalase and superoxide dismutase, and non-enzymatic antioxidants comprise mannitol, glutathione and carotenoids [20]. In addition to ROS, reactive nitrogen species (RNS), such as nitric oxide, may be involved in coral bleaching [18, 21]. Increased levels of nitric oxide in Symbiodiniaceae cultures and the sea anemone *Exaiptasia diaphana*, and increased activities of nitric oxide-producing enzymes in Symbiodiniaceae have been correlated with cnidarian bleaching and thermal stress [22–25]. Several studies suggest that nitric oxide plays a role in inducing host apoptotic pathways in response to symbiont dysfunction during bleaching [25–28]. Signalling pathways of ROS and RNS may also interact [18, 22, 26]. One interaction is the generation of peroxynitrite (ONOO^−^) from O_2_^−^ and nitric oxide, which disrupts electron transport within mitochondria [21] and has been linked to thermal stress in Symbiodiniaceae [26].

Aside from the oxidative stress theory which poses that the overproduction of ROS and RNS caused by light and temperature stress triggers a cellular cascade resulting in bleaching, some studies postulate that the bleaching cascade is triggered by the host’s inability to provide enough CO_2_ to the faster growing Symbiodiniaceae under increased temperatures [29, 30]. This is believed to disrupt the Calvin-Benson cycle and result in an overproduction of ROS by Symbiodiniaceae, which may leak into the host cells and initiate the bleaching cascade. A third theory is that elevated temperatures affect nutrient cycling between the coral and its algal symbiont [2]. Heat stress increases host respiration and catabolic processes, resulting in ammonium becoming available to the Symbiodiniaceae. Consequently, Symbiodiniaceae are freed from their normally nitrogen-limited state permitting their growth to increase and resulting in them using most of their photosynthate for their own growth rather than translocating it to the coral host. The Symbiodiniaceae quickly run out of phosphorus and the ensuing N:P imbalance is believed to cause a change in the composition of their thylakoid membranes and impair the photosystem [31], creating again an overproduction of ROS which may trigger the loss of the Symbiodiniaceae from the coral host.

Based on the roles of ROS and RNS in coral bleaching, mechanisms that neutralise these molecules may boost coral heat tolerance. Indeed, corals and coral model animals (sea anemones) bleached less when ROS levels were decreased through the addition of exogenous antioxidant compounds [32, 33], and a nitric oxide scavenging compound mediated decreases in photosynthetic efficiency of Symbiodiniaceae under heat stress [25]. We hypothesise that enhancing ROS and RNS-degradation within the coral holobiont by microbiome manipulation, such as inoculating corals with bacteria that have a high ROS and/or RNS-scavenging ability, may be a useful conservation strategy. While this may reduce the expulsion of algal symbionts, the coral host is likely still carbon-limited due to higher respiration rates and lower carbon translocation from the Symbiodiniaceae. Thus, ROS and RNS-scavenging bacteria that have the additional potential to translocate carbon to the host may provide an added benefit by minimising host starvation [34]. The presence of sugar transporters that can translocate carbon from the bacterial cell is therefore a likely beneficial trait. The “sugars will eventually be exported transporter” (SWEET) found in plants and other eukaryotes [35, 36] can bidirectionally transport small sugar molecules, in particular glucose [37]. SemiSWEET proteins are the bacterial homologues of SWEET proteins.

Here, we identify bacterial probiotic candidates to mitigate thermal stress in the scleractinian coral *Galaxea fascicularis*, which is an emerging coral model [38]. We analysed the genome sequences of 82 pure-cultured bacterial strains isolated from *G. fascicularis* with a focus on traits involved in ROS and RNS-scavenging, and sugar export mechanisms. Further, we surveyed for a range of other potentially beneficial metabolic pathways and genomic features that may indicate a stable host association, such as secretion systems that have been found in mutualistic endosymbionts and are known to facilitate evasion of eukaryotic host immune systems. Further, eukaryote-like repeat proteins (ELPs), including microbial ankyrin-repeat proteins (ARPs) and WD40-repeats, are suggested to facilitate host infection and generally promote stable symbiosis by assisting protein–protein interactions [39, 40].

## Materials and methods

### Culturing and identification of *G. fascicularis*-associated bacterial isolates

Three colonies of the coral *G. fascicularis* were collected from Sudbury Reef (S -17° 01 E 14° 21), Great Barrier Reef, Australia, brought into the aquaria facilities of Cairns Marine (Cairns, Queensland) and then shipped to the University of Melbourne in February 2020. After arrival, the corals were kept overnight in a 140-L recirculating aquarium system containing reverse osmosis water reconstituted Red Sea Salt™ (RSS, R11065, Red Sea, USA) at 26 ± 1.1 °C and at a salinity of 35 ± 0.5 parts per thousand (ppt). For bacterial culturing and 16S rRNA gene metabarcoding, coral tissue and mucus of a total of 30 randomly selected polyps per colony were sampled in 100 mL of filter-sterilised (0.2 µm) RSS water (fRSS) using a water flosser (Waterpik, Australia). Tissue homogenates were centrifuged in sterile Falcon tubes at 3750 rcf for 10 min and resulting tissue pellets were transferred into 1.5-mL sterile Eppendorf tubes and centrifuged at 5000 rcf for another 10 min. After removing the supernatant, tissue pellets were homogenised in 1-mL fRSS by using a tissue lyser at 30 Hz for 30 s (Tissue-Lyser II, Qiagen, Chadstone, Australia). Afterwards, serial dilutions from 10^−2^ to 10^−7^ of each homogenate per colony were created. The remaining undiluted tissue homogenate (4 times 200 µL) was transferred to 1.5-mL sterile Eppendorf tubes, flash frozen in liquid nitrogen and stored in − 80 °C until further processing for 16S rRNA gene metabarcoding. Fifty microliters of each dilution was spread plate-inoculated onto three petri dishes of R2A (R2A Agar CM0906, Oxoid Ltd. Basingstoke, Hampshire, England), which was complemented with 40 g L^−1^ fRSS, and onto three petri dishes of MA (Difco™ Marine Agar 2216, BD, Sparks, MD, USA). Petri dishes were incubated in the dark at 26 °C, which resembled the seasonal seawater temperature of the collection site. After 1 week of incubation, individual colonies were picked and each colony was sub-cultured on new media until purity. Individual colonies (presumably comprised of one bacterial strain) were resuspended in 200 µL of 40% glycerol and stored at − 80 °C.

To identify *G. fascicularis*-sourced isolates, freshly grown bacterial colonies were lysed in 20 µL Milli-Q water at 95 °C, centrifuged at 2000* g* for 1 min and supernatants were used as DNA templates for subsequent colony PCR amplification. Colony PCRs were conducted using the bacterial primers 27f (50- AGA GTT TGA TCM TGG CTC AG-30) and 1492r (50- TAC GGY TAC CTT GTT ACG ACT T-30) [41] following a protocol previously described [42] but with modified thermal cycles as follows: 5 min at 94 °C; 30 cycles of 60 s at 94 °C, 45 s at 50 °C and 90 s at 72 °C; 10 min at 72 °C; with a final holding temperature of 4 °C. After verifying the generation of 16S rRNA gene amplicons by 1% agarose gel electrophoresis, PCR products were purified and Sanger sequenced on an ABI sequencing machine using the primer 1492r at Macrogen (Seoul, South Korea). Raw sequences were aligned and trimmed in Geneious Prime 2021.1.1 (https://geneious.com), and the corrected 16S rRNA gene sequences (~ 1000 bp) were compared via BLASTN (https://blast.ncbi.nlm.nih.gov) to GenBank sequences to find the highest percent identities.

### DNA extraction and data processing for 16S rRNA gene metabarcoding

To assess how well the bacterial isolates represent the full *G. fascicularis* bacterial microbiome, we performed amplicon sequencing of the 16S rRNA gene. We extracted the DNA from three tissue homogenate samples per coral colony of *G. fascicularis* according to established protocols from the Marine Microbial Symbiosis Lab (The University of Melbourne) [43] and included negative controls (*n* = 3) that did not contain samples to determine contaminants from DNA extractions. Extracted DNA and negative controls (3 DNA extraction controls, 3 PCR controls) were amplified in triplicates and sequencing libraries were created, both following established protocols [44]. Libraries were sequenced at the Walter and Eliza Hall Institute of Medical Research (WEHI, The University of Melbourne) in one Illumina Miseq run using v3 (2 × 300 bp) reagents.

Sequencing resulted in an average of 40,786 reads per sample. The 16S rRNA gene sequences were processed and demultiplexed in QIIME2 v2021.4 [45]. Cutadapt v2.6 was used to remove primers [46]. Quality control and filtering steps were conducted in DADA2 [47] in the QIIME2 environment, which included correction of sequencing errors, removal of low-quality bases (average Qscore < 30) and generation of amplicon sequence variants (ASVs). Quality control and filtering steps resulted in 20,244 reads per sample and a total of 3154 ASVs were determined. Taxonomy of each ASV was assigned using the SILVA v138 database [48]. Diversity metrices of 16S rRNA gene metabarcoding datasets were analysed in R [49] using *phyloseq* [50] and *microbiome* [51] packages.

### Genomic DNA extraction and whole-genome sequencing of bacterial isolates

Eighty-two out of 91 bacterial isolates were selected for whole-genome sequencing with the aim of covering as much taxonomic diversity as possible. Genomic DNA of freshly grown bacterial cultures was extrac ted using the Invitrogen™ PureLink™ Genomic DNA Mini Kit (K182001, Invitrogen™, Thermo Fisher Scientific, Australia) according to the manufacturer’s protocol but including the following modifications: 10 mg mL−1 lysozyme was added to the lysozyme digestion buffer, samples were sonicated for 2 min (POWERSONIC 505 Digital Ultrasonic Bath, Thermoline Scientific, Australia) after resuspending the cell pellet in in the lysozyme digestion buffer, and 20 µL of RNase (supplied by the kit) was added after incubating the samples at 55 °C. DNA concentrations were assessed using the Quant-iT™ PicoGreen™ dsDNA Assay Kit (P7589, Invitrogen™, Thermo Fisher Scientific, Australia), following the manufacturer’s guidelines and measuring its absorbance at 485 nm excitation and a wavelength of 530 nm (CLARIOstar PLUS plate-reader, BMG Labtech, Australia). The quality of the extracted DNA was determined using UV5Nano (Mettler Toledo, Australia). In preparation for whole-genome sequencing, the concentration of the extracted genomic DNA of each bacterial isolate was adjusted to 20 ng/µL. Multiplexed Nextera XT (Illumina) libraries were sequenced at Doherty Applied Microbial Genomics (Doherty Institute, The University of Melbourne, Australia) on the Illumina NextSeq550 platform, creating 150 bp paired-end reads.

### Genome assembly and annotation

Raw reads were trimmed and quality-filtered (removed first 10 bases, reads < 30 bp and reads with a Phred score ≤ 28 over the average of 4 bp) using Trimmomatic v0.39 [52]. The quality of raw reads before and after trimming was checked with FASTQC v0.11.5 [53] and collated using MultiQC v1.12 [54]. Trimmed and quality-filtered reads were de novo assembled into contigs using SPAdes genome assembler 3.11.1 [55] with 21, 33, 55 and 77 k-mers and the option “–careful” was applied to minimise the number of mismatches and short indels. All contigs with a length of < 1000 bp were removed using BBMap v38.96 [56]. Subsequently, the levels of completeness, heterogeneity and contamination of assembled genomes were assessed using CheckM v1.16 [57]. Gene prediction was performed in Prokka v1.14.6 [58] which uses prodigal v2.6.3 [59] with following parameters “—addgenes”, “—addmrna”, “—rfam”. Predicted proteins were subjected to Interproscan 5.55 v88.0 [60] search to annotate protein family (Pfam) ids using with the parameter “-appl pfam” and evalue < 1e − 5. Taxonomic assignment of all assembled genomes was carried out based on the Genome Taxonomy Database (GTDB) [61] via GTDB-Tk v2.1.0 [62] using the “classify_wf” workflow. GTDB-Tk assigned the taxonomy of the genomes based on 120 bacterial marker genes. A phylogenetic tree was built in IQ tree v1.6.1 [63], using the maximum likelihood methods with a LG + F + R7 model (general matrix (LG), empirical base frequencies (F), FreeRate model with 7 categories (R7), AIC 424879.963, BIC 426067.398)) which was selected as the best model by ModelFinder Plus as implemented in IQ tree, and using 1000 ultrafast bootstraps from multiple sequence alignments of 120 bacterial marker genes generated by GTDB-Tk. The tree was visualised in iTOL v6 [64].

### Identification of genomic features, pathways and genes of interest

Genomic features, including average nucleotide identity (ANI), average amino acid identity (AAI) and in silico genome-genome distance (GGD), were calculated using default parameters from FastANI v1.33 [65], FastAAI (https://github.com/cruizperez/FastAAI) and GenDisCal v1.3.1 (https://github.com/LM-UGent/GenDisCal), respectively. All matrices depicting ANI, AAI and GGD were plotted in R v1.4.17 [49] using the package “ggplot2” v3.3.5 [66]. Metabolic pathways, transport systems and secretion systems were annotated via METABOLIC-G v4.0 [67] applying “-m-cutoff 0.50” to include pathways which are ≥ 50% complete. The completeness of metabolic pathways (KEGG module database, https://www.genome.jp/kegg/module.html), transporters and secretion systems was estimated in EnrichM v0.6.4 [68]. Metabolic pathways and transporters, and secretion systems that were found to be ≥ 80% complete for one or more isolates were selected and plotted using the package ggplot2 v3.3.5 [66] in R (Supplementary Fig. 2). Interproscan 5.55 v88.0 [60] was used to annotate different categories of ELPs and other genes of interest using Pfam IDs (Table 1). All Pfam identification codes were acquired from the Uniprot database (https://www.uniprot.org/uniprotkb). The presence of ARP and WD40 ELP families was identified based on their Pfam identification codes (Table 1). SemiSWEET transporters were also detected by their Pfam identification codes (Table 1). We aligned the protein sequences of each semiSWEET transporter found in the isolates using MUSCLE v3.8.425 [69] within Geneious Prime v2021.1.1. A phylogenetic tree of the alignment of semiSWEET proteins was built in IQ tree v1.6.1 [63] applying a mtZOA + G4 model (Mitochondrial Metazoa protein model (mtZOA), Gamma rate heterogeneity (G), AIC 3359.763, BIC 3407.600) selected by ModelFinder Plus and visualised in iTOL v6 [64]. Multiple genes involved in ROS and RNS-scavenging were also identified based on their Pfam identification codes (Table 1). A gene involved in ROS-scavenging included *gshAB* (bifunctional glutamate-cysteine ligase *gshA*/glutathione synthetase *gshB*), which is responsible for the biosynthesis of the antioxidant glutathione. The synthesis of the antioxidant β-carotene was detected by the presence of gene *crtY* (lycopene β-cyclase), while the synthesis of the antioxidant zeaxanthin from β-carotene was identified by the presence of gene *crtZ* (β-carotene hydroxylase). Genes encoding the biosynthesis of the antioxidant mannitol were determined via mannitol-1-phosphate dehydrogenase (*mtlD*) and mannitol 2-dehydrogenase (*mtlk*). The enzymatic superoxide scavenging potential was identified via the presence of superoxide dismutase (SOD). Biosynthesis of the antioxidant dimethylsulphide (DMS) via the cleavage of dimethylsulfoniopropionate (DMSP) was determined by the presence of DMSP lyase (*dddQ, dddY, dddL*), and the biosynthesis of the antioxidant DMSP via the presence of *dsyB* (methylthiohydroxybutyrate methyltransferase). The potential to scavenge different forms of RNS, such as the reduction of peroxynitrite, was inferred from the presence of peroxiredoxin (*ahpC*). Scavenging of nitric oxide was assessed via two different genes, *hmp* (nitric oxide dioxygenase) and *norBC* (nitric oxide reductase subunits C (*norC*) and B (*norB*)). We also screened for the gene responsible for converting nitrous oxide (product of *norBC*) to nitrogen and completing denitrification, *nosZ* (nitrous oxide reductase).

**Table 1:** Genes and respective protein families (Pfam IDs) for which the *G. fascicularis*-associated bacterial genomes were interrogated. Pfam IDs of interest were chosen based on their known involvement in ROS and RNS-scavenging via enzymatic activity or the production of key antioxidants, translocation of sugars via bidirectional sugar transporters and establishment and promotion of the coral-bacteria symbiosis through eukaryotic repeat proteins. Pfam IDs of interest were sourced from the Uniprot database (https://www.uniprot.org/uniprotkb). To annotate Pfam IDs of interest Interproscan 5.55 v88.0 [60] was used

	**Function**	**Gene/Protein**	**Protein Family (PFAM) ID**
**ROS-scavenging**	Glutathione synthesis	*gshAB*	PF04262, PF02955, PF02951
	Zeaxanthin and β-carotene synthesis	*crtZ, crtY*	PF04116, PF18916
	Mannitol synthesis	*mtlD, mtlK*	PF01232, PF08125
	Superoxide scavenging (Superoxide dismutase)	SOD MnFe	PF00081, PF2777
	Dimethyl sulphide synthesis	*dddQ, dddY, dddL*	PF16867
	Dimethylsulfoniopropionate synthesis	*dsyB*	PF00891, PF16864
**RNS-scavenging**	Peroxynitrite reduction	*ahpC*	PF10417
	Nitric oxide reduction	*hmp*	PF00970, PF00175, PF00042
	Nitric oxide reduction	*norB, norC*	PF00115, PF00034
	Nitrous oxide reduction	*nosZ*	PF18764, PF18793
**Carbon translocation**	Bidirectional sugar transport	SemiSWEET protein	PF04193, PF03083
**Symbiosis establishment/promotion**	Eukaryote-like repeat proteins	Ankyrin-repeat proteins	PF00023, PF12796, PF13637, PF13857
		WD40-repeat proteins	PF00400, PF07676

## Results

### Diversity of *G. fascicularis*-associated cultured bacteria

**Fig. 1:**
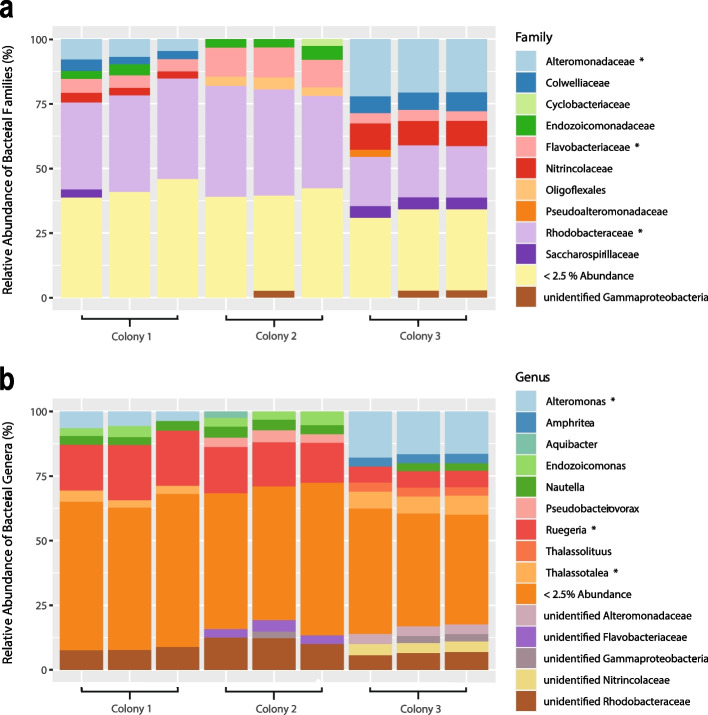
Bacterial community composition (based on 16S rRNA gene metabarcoding) of *G. fascicularis* colonies used for culturing highlighting **a** the most abundant bacterial families and **b** the most abundant bacterial genera of each replicate sample per colony of *G. fascicularis* (*n* = 3 per coral colony; SILVA database v. 138). The three most abundant families/genera are highlighted using asterisks. Less abundant bacterial families or genera are grouped in one category (< 2.5% relative abundance)

In total, we cultured 613 bacterial isolates from the tissue and mucus of three Great Barrier Reef (GBR) colonies of *G. fascicularis*, spanning 54 genera and 91 species (Supplementary Table 1). Each bacterial isolate was first taxonomically identified by Sanger sequencing of the 16S rRNA gene and by choosing the closest match from the Basic Local Alignment Search Tool (BLASTn) [70]. Most of the isolates belonged to the classes Gammaproteobacteria (235 isolates, Supplementary Table 1), followed by Alphaproteobacteria (124), Flavobacteriia (27) and the genera *Bacillus* (180), *Alteromonas* (153), *Vibrio* (70) and *Ruegeria* (50). The genera to which these bacterial isolates belong accounted for 48% of the relative abundance in the bacterial communities from the three *G. fascicularis* colonies (Fig. 1, Supplementary Fig. 1). Importantly, 70% of the genera to which the 17 most abundant amplicon sequence variants (ASVs) belong were obtained in pure culture, including *Ruegeria* and *Alteromonas*. Bacterial genera that were identified from metabarcoding to be among the most abundant ASVs in *G. fascicularis* colonies used for culturing, but which were not obtained in culture, included *Endozoicomonas* and *Thalassotalea*. The 17 most abundant ASVs were selected based on their relative abundance of > 2%.

**Fig. 2:**
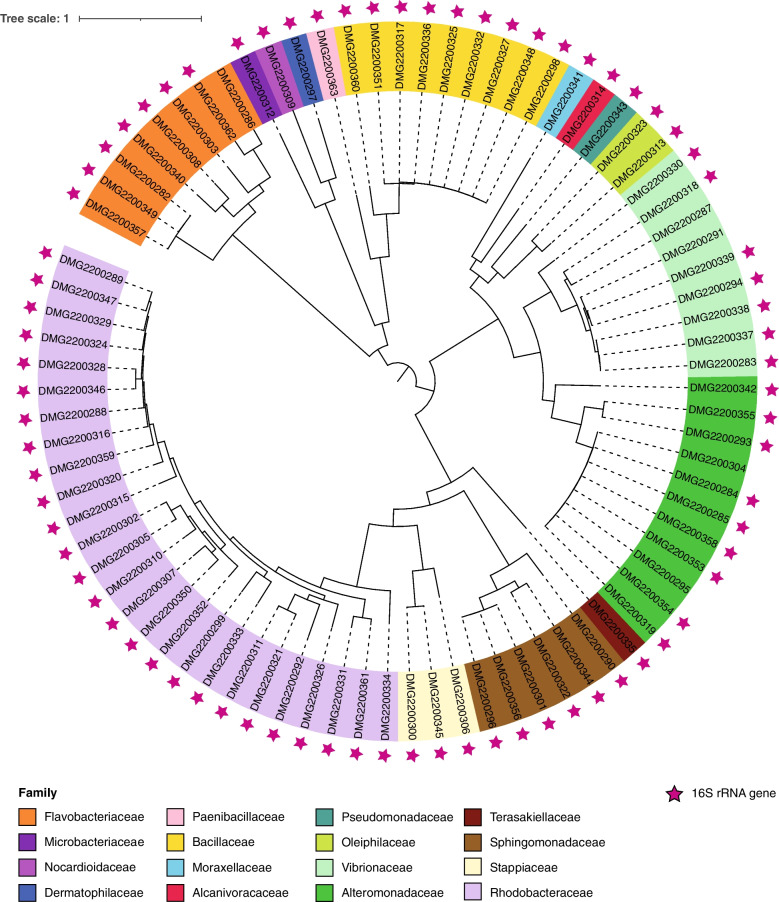
Phylogenetic tree of the 82 pure-cultured *G. fascicularis*-associated bacteria. The 82 isolates were assigned to 16 bacterial families. The 16S rRNA genes were assembled (pink stars) for all but five genomes. Taxonomic assignment of the bacterial genomes was conducted using the Genome Taxonomy Database (GTDB) [61] via GTDB-Tk v2.1.0 [62] based on 120 bacterial marker genes. The phylogenetic tree was constructed using the maximum likelihood method in IQ tree v1.6.1 [63] with a LG + F + R7 model selected as the best model by ModelFinder Plus, and using 1000 ultrafast bootstraps from multiple sequence alignments of the 120 bacterial marker genes. The tree was visualised in iTOL v6 [64]. DMG = Doherty Microbial Genomics

We sequenced and assembled genomes of 82 isolates spanning 16 bacterial families including Rhodobacteraceae (GTDB-Tk classification, 26 isolates) and Alteromonadaceae (11) (Fig. 2). Overall, 37 different bacterial genera were identified via GTDB-Tk using 120 bacterial marker genes (Supplementary Table 2). The most abundant genera in the collection were *Alteromonas* (10 isolates), *Ruegeria* (10), *Bacillus* (8), *Vibrio* (8) and *Qipengyuania* (5, basionym: *Erythrobacter*, Supplementary Table 2). When comparing the taxonomic assignment of the bacterial genomes via GTDB-Tk with the taxonomic assignment of the 16S rRNA gene sequences of the isolates via the closest hit in NCBI, fewer genera were assigned to the bacterial genomes (37 genera in 82 isolates (120 marker genes; Supplementary Table 2) compared to 54 genera in 91 isolates (16SrRNA gene; Supplementary Table 1)). This discrepancy stems from the fact that the assigned genera vary in some cases between GTDB and 16S rRNA gene sequences (closest BLASTn hit in NCBI) and from small differences in taxonomic nomenclature between GTDB and NCBI.

### Characteristics of assembled bacterial genomes

All 82 assembled bacterial genomes exhibited an average completeness of 99.59 ± 0.45% and a contamination of 0.46 ± 0.41% (Supplementary Table 2, Supplementary Figs. 4–6). Genome sizes averaged 4.34 ± 0.86 Mb. On average, these genomes were assembled into 54.47 ± 41.57 contigs, encoded 4071.96 ± 754.07 genes and had a coding density of 89.57 ± 1.96%. Guanine-cytosine (GC) content averaged at 51.67 ± 9.88% across all genomes, and 16S rRNA genes were assembled in 77 genomes (Fig. 2).

### Presence of genes, metabolic pathways and genomic features of interest in *G. fascicularis*-associated bacterial isolates

**Fig. 3:**
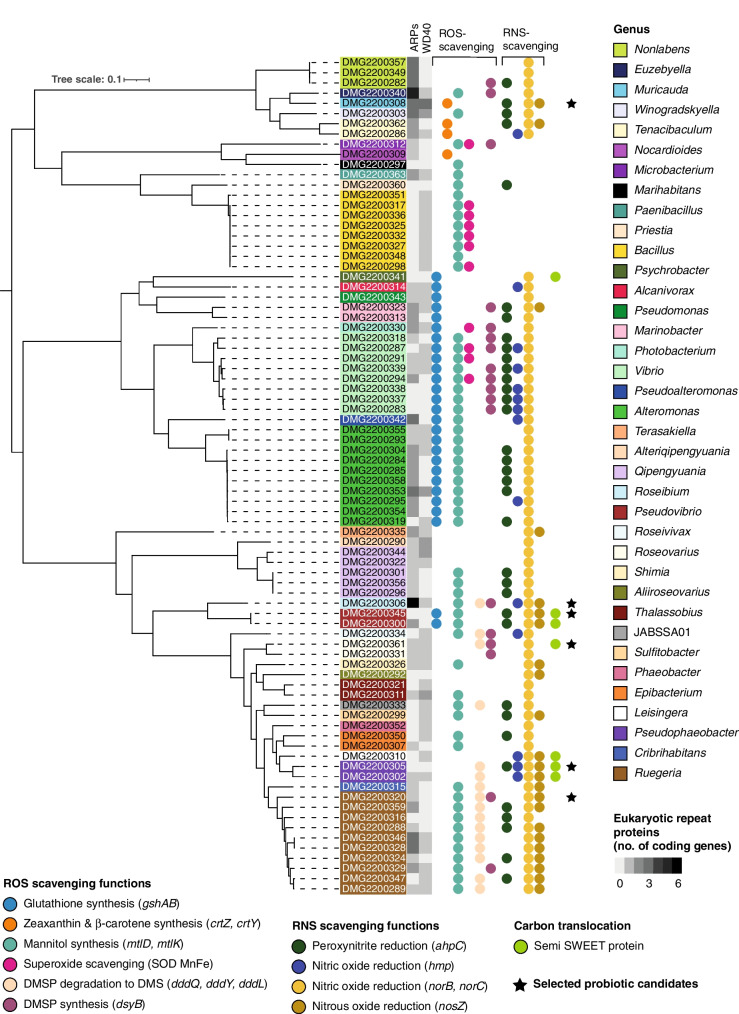
Presence of ROS and RNS-scavenging genes, semiSWEET proteins and eukaryotic repeat proteins (ARPs and WD40-repeats), shown in a phylogenetic tree of 82 *G. fascicularis*-associated bacteria. The 82 isolates were assigned to 37 bacterial genera. Selected probiotic candidates are indicated with a black star. Taxonomic assignment of the bacterial genomes was conducted using the Genome Taxonomy Database (GTDB) [61] via GTDB-Tk v2.1.0 [62] based on 120 bacterial marker genes. The phylogenetic tree was constructed using the maximum likelihood method in IQ tree v1.6.1 [63] with a LG + F + R7 model selected as the best model by ModelFinder Plus, and using 1000 ultrafast bootstraps from multiple sequence alignments of the 120 bacterial marker genes. The tree was visualised in iTOL v6 [64]. DMG = Doherty Microbial Genomics

Annotated genomes were screened for genes with roles in ROS and RNS-scavenging (Table 1, Fig. 3, Supplementary Table 2). Genes of interest involved in glutathione, mannitol and DMSP synthesis were found across a diverse range of bacterial families, whereas genes for the synthesis of zeaxanthin, β-carotene and DMS, and superoxide scavenging were limited to two to three bacterial families (Fig. 3). All genes of interest with RNS-scavenging functions were detected across a large diversity of families (Fig. 3).

**Fig. 4:**
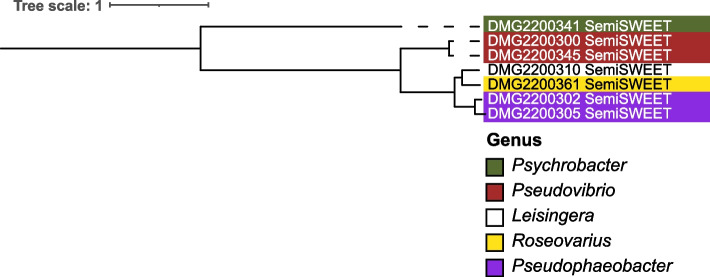
Phylogenetic tree of SemiSWEET protein sequences found in *G. fascicularis*-associated bacteria. Protein sequences of each semiSWEET transporter were aligned using MUSCLE v3.8.425 [69] within Geneious Prime v2021.1.1. The phylogenetic tree was constructed using the maximum likelihood method in IQ tree v1.6.1 [63] with a mtZOA + G4 model selected as the best model by ModelFinder Plus. The tree was visualised in iTOL v6 [64]. DMG = Doherty Microbial Genomics

Metabolic pathways (KEGG pathway modules) that were found to be ≥ 80% complete in one or more bacterial isolates include amino acid metabolism, biosynthesis of terpenoids and polyketides, carbohydrate metabolism, energy metabolism, glycan metabolism, lipid metabolism, metabolism of cofactors and vitamins and nucleotide metabolism (Supplementary Fig. 2, Supplementary Table 3, Supplementary results text “Complete metabolic pathways identified in *G. fascicularis*-associated bacteria”). Pathways that were linked to energy metabolism such as carbon metabolism were found to be 100% complete in several isolates. For instance, the reductive pentose phosphate cycle (KEGG module ID M00167) was shown to be 100% complete in Rhodobacteraceae (25 isolates), Sphingomonadaceae (5) and Stappiaceae (3, Supplementary Fig. 2, Supplementary Table 3). Another pathway involved in carbon metabolism, the phosphate acetyltransferase-acetate kinase pathway (M00579), was 100% complete in Rhodobacteraceae (13 isolates), Alteromonadaceae (9), Bacillaceae (9), Vibrionaceae (9), Microbacteriaceae (1), Moraxellaceae (1), Oleiphilaceae (1), Paenibacillaceae (1) and Pseudomonadaceae (1). Only four isolates contained an anoxygenic photosystem II (M00597), i.e. *Roseibium* sp. strain Doherty Microbial Genomics (DMG)2200306, *Roseivivax marinus* (DMG2200334) and two isolates of *Roseovarius* sp. (DMG2200331, DMG2200361). Pathways involved in nitrogen metabolism, such as denitrification (M00529), were 100% complete in Rhodobacteraceae (10 isolates), Stappiaceae (3) and Terasakiellaceae (1). Genes for the SemiSWEET protein were present in seven bacterial isolates (Fig. 3): *Psychrobacter* sp. (DMG2200341), two isolates of *Pseudovibrio denitrificans* (DMG2200345, DMG2200300), *Roseovarius* sp. (DMG2200361), *Leisingera* sp. (DMG2200310) and two isolates of *Pseudophaeobacter* sp. (DMG2200305, DMG2200302). Phylogenetic analysis based on an alignment of the semiSWEET proteins reflected the phylogeny of the bacterial isolates in which they were identified (Fig. 4).

Of the ELP proteins, we found ARPs to be more abundant than WD40-repeat proteins (Fig. 3). The greatest number of ARPs overall was found in *Roseibium* sp. (DMG2200306, 6 ARPs) and *Euzebyella* sp. (DMG2200340, 5 ARPs). The most WD40-repeat proteins were detected in *Muricauda* sp. (DMG2200308, 3 WD40-repeats). Secretion systems that were 100% complete comprised type II (T2SS), III (T3SS), IV (T4SS) and VI (T6SS, Supplementary Fig. 3). T2SS were well represented in five families, i.e. Alteromonadaceae (11 isolates), Vibrionaceae (9), Oleiphilaceae (2), Pseudomonadaceae (1) and Alcanivoraceae (1). T3SS were only detected in five isolates of Vibrionaceae, T4SS in *Vibrio harveyi* and six isolates of Rhodobacteraceae, and T6SS in Vibrionaceae (7), Stappiaceae (3), Oleiphilaceae (2), Alteromonadaceae (1) and Pseudomonadaceae (1).

### Selection of probiotic bacterial candidates

We selected six probiotic candidates (Fig. 3, black stars) by prioritising the presence of genes encoding ROS and/or RNS-scavenging, followed by the presence of semiSWEET transporters. Other selection criteria entailed genomic features that are suggested to enhance colonisation and association of bacteria with the coral host, i.e. ELPs (ARPs and WD40-repeats) and secretion systems. We excluded Vibrionaceae strains from the probiotic selection since this family is known to contain several coral pathogens [71].

One selected candidate, *Ruegeria* sp. (DMG2200320; GTDB-Tk classification; 98.03% of 16S rRNA gene sequence identity with *R. marisrubri* strain ZGT118 in NCBI), exhibits genes involved in mannitol (*mtlD, mtlK*), DMS (*dddQ*) and DMSP synthesis (*dsyB*, Fig. 3), including a transporter for mannitol (M00200, Supplementary Table 3). Both genes of interest encoding nitric oxide reduction (*hmp, norBC*) are also found in *Ruegeria* sp. DMG2200320, contributing to a complete denitrification pathway (M00529, Supplementary Fig. 2, Supplementary Table 3). DMG2200320 does not exhibit any secretion systems but has one ARP (Fig. 3). The complete cobalamin (vitamin B_12_) synthesis pathway is also present (M00122, Supplementary Fig. 2, Supplementary Table 3).

A second probiotic candidate is *Muricauda* sp. (DMG2200308; GTDB-Tk classification; 99.04% 16S rRNA gene sequence identity with *M. ruestringensis* DSM 13258 in NCBI), which contains both genes for zeaxanthin and β-carotene synthesis (*crtZ, crtY*), as well as for the degradation of peroxynitrite (*ahpC*), nitric oxide (*norBC*) and nitrous oxide (*nosZ*, Fig. 3). DMG2200308 also displays three ARPs and WD40-repeat proteins each, which was the highest number of WD40-repeats recorded across all isolates (Fig. 3).

A third probiotic candidate, *Roseibium* sp. (DMG3300306; GTDB-Tk classification; 98.91% 16S rRNA gene sequence identity with *R. album* strain 5OM6 in NCBI; homotypic synonyms *Stappia alba* [72] and *Labrenzia alba* [73]), contains the genes for mannitol (*mtlD, mtlK*), DMS (*dddQ*) and DMSP (*dsyB*) synthesis (Fig. 3) and a transporter for mannitol (M00200, Supplementary Table 3). We also detected genes for the reduction of nitric oxide (*norBC, hmp*) and nitrous oxide (*nosZ*, Fig. 3), and the complete denitrification pathway (M00529, Supplementary Fig. 2, Supplementary Table 3). DMG3300306 presents the highest number of ARPs among all isolates (6 ARPs), one WD40-repeat protein (Fig. 3) and a complete T6SS (Supplementary Fig. 3).

A fourth probiotic candidate, *Roseovarius* sp. (DMG2200361; GTDB-Tk classification; 98.8% 16S rRNA gene sequence identity with *R. aestuarii* strain SMK-122 in NCBI), contains genes for the synthesis of DMSP (*dsyB*) and the reduction of nitric oxide (*norBC*, Fig. 3). DMG2200361 also contains a semiSWEET sugar transporter (Fig. 3). One ARP and WD40-repeat protein each was detected in *Roseovarius* sp. DMG2200361 (Fig. 3), as well as one complete T4SS (Supplementary Fig. 3).

A fifth selected probiotic candidate *Pseudophaeobacter* sp. (DMG2200305; GTDB-Tk classification; 96.1% 16S rRNA gene sequence identity with *Leisingera methylohalidivorans* DSM 14336 strain MB2 in NCBI) is the only strain to display all genes for RNS-scavenging (*ahpC, hmp, norBC, nosZ*) and *dddQ* for the synthesis of DMS (Fig. 3). We also found a semiSWEET transporter (Fig. 3) in strain DMG2200305. We did not identify any ELPs in *Pseudophaeobacter* sp. DMG2200305, but a complete T4SS (Supplementary Fig. 3).

*Pseudovibrio denitrificans* (DMG2200345; GTDB-Tk classification; 100% 16S rRNA gene sequence identity with *P. denitrificans* strain DN34 in NCBI) is the sixth probiotic candidate we selected and possesses genes for the synthesis of antioxidants glutathione (*gshAB*) and mannitol (*mtlD*, *mtlK*, Fig. 3). RNS-scavenging genes in DMG2200345 include peroxynitrite (*ahp*C), nitric (*nosBC*) and nitrous oxide (*norZ*) reduction (Fig. 3), and a complete denitrification pathway (M00529, Supplementary Fig. 2, Supplementary Table 3). We discovered a semiSWEET transporter (Fig. 3) and a T6SS, but not any ELPs (Supplementary Fig. 3) in *P. denitrificans* DMG2200345.

## Discussion

To identify probiotic bacterial candidates that may assist in building coral bleaching resilience, we examined the functional potential of *Galaxea fascicularis*-associated bacteria via genomic screening. We identified *G. fascicularis*-associated bacteria with various novel combinations of putative beneficial functions, such as ROS and/or RNS-scavenging that may mitigate thermal stress in the coral holobiont, carbon translocation which might aid bleaching resistance and recovery, and genomic features suggested to enhance bacterial colonisation of and association with the coral holobiont. In *G. fascicularis*, ROS is believed to be a major driver of thermal bleaching [74], and the selected probiotic candidates may neutralise ROS and reduce or prevent bleaching in this coral species. Our selection of bacterial probiotic candidates may also be relevant for a broad range of other coral species as they are taxa commonly found in scleractinian corals. Further, *G. fascicularis* is known to be widespread across a broad spectrum of reef environments worldwide and even is the dominant species on some inshore fringing reefs [75].

### Whole-genome sequenced bacterial isolates represent *G. fascicularis*-associated bacterial diversity

The 82 bacterial isolates for which genomes were obtained comprise 16 families and 37 genera. These families make up the majority of the bacterial microbiome associated with the three GBR-sourced *G*. *fascicularis* colonies. The most abundant genera that were identified by 16S rRNA gene metabarcoding, *Ruegeria* (11.68% average relative abundance) and *Alteromonas* (7.73%), were also obtained in pure culture. Bacteria that were found in the *G. fascicularis* bacterial microbiome but which we were not able to culture included genera such as *Endozoicomonas*, which has gained increased attention as a potential indicator for coral health [76] and which has been shown to play a role in the coral sulphur cycle by metabolising DMSP to DMS [77]. In general, the microbiome of the GBR colonies is similar to that of *G. fascicularis* from the South China Sea [78, 79]. When expanding the focus to bacterial microbiomes of scleractinian corals in general, all bacterial classes in our culture collection (i.e. Gammaproteobacteria, Alphaproteobacteria, Bacilli and Flavobacteriia) have been found to be coral-associated [80, 81]. Taken together, our collection of bacterial genomes was a comprehensive representation of the bacterial diversity associated with *G. fascicularis* and scleractinian corals in general.

### Potential of *G. fascicularis*-sourced probiotic candidates to scavenge ROS and RNS

Three of the selected probiotic candidates, i.e. *Ruegeria* sp. DMG2200320, *Roseibium* sp. DMG3300306 and *Roseovarius* sp. DMG2200361, exhibit the potential of producing the two antioxidants DMS and DMSP [82]; *Pseudophaeobacter* sp. DMG2200305 contains genes for the production of DMS only. DMS synthesis via demethylation or cleavage of DMSP has previously been reported for strains of *Pseudophaeobacter* sp. [83], *Ruegeria* sp. [84, 85] and *Roseovarius* sp. [86], whereas DMSP biosynthesis via *dsyB* is novel for the latter two genera. Both DMS and DMSP synthesis are known for *Roseibium* sp. [87, 88]. Generally, DMSP and DMS are both considered effective antioxidants that scavenge OH^−^, with DMS being the more reactive compound [82]. DMSP and DMS can also act as a carbon and sulphur source for microbes [89] or can shape coral microbial communities via antimicrobial properties [90]. DMSP can also act as a chemo-attractant for the coral pathogen *Vibrio coralliilyticus* to colonise the coral host [91]. We detected the potential to synthesise the antioxidant mannitol in three of the selected probiotic candidates (*Ruegeria* sp. DMG2200320, *Roseibium* sp. DMG3300306, *Pseudovibrio denitrificans* DMG2200345), which is a novel observation for *Ruegeria* sp. and *P. denitrificans*. Mannitol scavenges OH^−^ [92] and could mitigate thermal stress in corals. For instance, exogenous addition of mannitol to corals reduced Symbiodiniaceae loss in *Agaricia tenuifolia* during heat stress [32], and reduced DNA damage in *Pavona divaricate* host tissues during thermal stress [93]. It also mitigated bleaching in *E. diaphana* [94]*.* The synthesis of other antioxidants, zeaxanthin and β-carotene, was identified in the probiotic candidate *Muricauda* sp. DMG2200308, supporting previous findings about this genus [95, 96]. Carotenoids belong to the most potent antioxidants by quenching the highly reactive singlet oxygen [97, 98]. Zeaxanthin produced by *Muricauda* sp. strain GF1 mitigated light and thermal stress via the reduction of ROS in cultured Symbiodiniaceae [95]. Zeaxanthin produced by *Muricauda* sp. isolated from coastal marine sands was demonstrated to scavenge nitric oxide [96]. Thus, zeaxanthin is an antioxidant that might mitigate both ROS and RNS overproduction in the coral holobiont.

All selected probiotic candidates display nitric oxide reduction potential via *norBC*, whereas *Roseibium* sp. DMG3300306 and *Pseudophaeobacter* sp. DMG2200305 also contains *hmp* for this property. Furthermore, all candidates, except for *Roseovarius* sp. DMG2200361, show the potential to convert nitrous oxide to nitrogen. Nitric oxide (*norBC*) and nitrous oxide reductases (*nosZ*) were previously documented in *Ruegeria* [84], *Muricauda* [99], *Roseibium* [100], *Pseudovibrio* [101] and *Roseovarius* (*norBC* only) [102], while the discovery of *hmp* in *Roseibium* sp. DMG3300306 and *Pseudophaeobacter* sp. DMG2200305 is novel for these genera. The reduction of nitric oxide via probiotic bacteria might be advantageous for the coral under thermal stress, especially if targeting its algal symbionts, since the addition of a nitric oxide scavenging compound alleviated a decrease in photosynthetic performance in Symbiodiniaceae cultures under heat stress [25]. Three of the selected probiotic candidates also show the potential to reduce peroxynitrite to nitrate via *ahpC* (*Muricauda* sp. DMG2200308, *P. denitrificans* DMG2200345 and *Pseudophaeobacter* DMG2200305), which is a novel pathway for *Muricauda* and *Pseudophaeobacter*. A relevant observation from an earlier study is that *ahpC* from coral-associated *Bacillus aquimaris* protected *Escherichia coli* from oxidative stress [103]. The presence of all four RNS-scavenging features is novel for *Pseudophaeobacter*, calling for further studies testing their functionality.

### Probiotic candidates with carbon translocation potential

The ability to export carbon, especially glucose [104], to the coral host may be an advantageous trait for coral probiotic candidates, and may be used to provide energy to enhance bleaching tolerance and facilitate bleaching recovery of the carbon-starved host [34, 105]. This study provides the first report of any coral-associated bacteria possessing semiSWEET protein genes, potentially giving them the ability to export small sugar molecules just like the eukaryotic homologue SWEET, although sugar export has not yet been confirmed in bacteria [72]. SemiSWEET transporters were discovered, among others, in the selected probiotic candidates *Roseovarius* sp. DMG2200361, *Pseudophaeobacter* sp. DMG2200305 and *P. denitrificans* DMG2200345, novel findings for these genera, suggesting that these strains might export carbon to the coral host.

### Probiotic candidates possess putative traits for a stable host association

For bacterial probiotics to be a viable intervention to enhance coral climate resilience, long-term beneficial effects on the coral holobiont must be achieved [106]. It is therefore important that probiotic bacteria form a stable association with the holobiont. In this study, we identified proteins with ARPs and/or WD40-repeat proteins in four of the selected probiotic candidates (*Ruegeria* sp. DMG2200320, *Muricauda* sp. DMG2200308, *Roseibium* sp. DMG3300306 and *Roseovarius* sp. DMG2200361). These ELPs have been hypothesised to promote stable symbiotic associations through bacterial protein-eukaryotic host protein interactions, as indicated for a range of coral-associated bacteria from *Porites lutea* [107] and the suggested coral bacterial symbiont *Endozoicomonas* sp. [77]. *Muricauda* sp. DMG2200308 and *Roseibium* sp. DMG3300306 exhibited the highest numbers of ARPs and WD40-repeats among *G. fasciularis*-associated bacterial isolates, and we hypothesise that those may facilitate symbiotic interactions with the coral host and Symbiodiniaceae. T4SS, detected in *Roseovarius* sp. DMG2200361 and *Pseudophaeobacter* sp. DMG2200305, might also assist in their association with the coral holobiont by translocating ankyrin-repeat-containing effectors, a mechanism that has been reported for a range of bacteria [40]. One study suggested that T4SS found in *Roseovarius mucosis* aids colonisation of its dinoflagellate host *Alexandrium ostenfeldii* [108]. T6SS, found in *Roseibium* sp. DMG3300306 and *P. denitrificans* DMG2200345, is a commonly found secretion system in bacteria that plays a role in virulence and antibacterial activity [109] and might promote bacterial communication [110]. T6SS in *Vibrio fischeri* has been described to play a role in the establishment of the symbiosis with the bobtail squid via eliminating bacterial competitors [111]. This secretion system has also been detected in the coral bacterial symbiont *Endozoicomonas* sp. [112]. Whether both T6SS and T4SS, which are the only secretion systems (including T3SS) that can transport proteins across an extra host cell membrane [113], may play beneficial roles in the establishment of the discussed probiotic candidates with the coral host and/or Symbiodiniaceae requires further studies.

Some of the selected probiotic candidate genera isolated in this study are known to form stable associations with corals, such as *Ruegeria* spp. [114]. For example, members of *Ruegeria* have been observed in both early and adult life stages of *Pocillopora damicornis* [115]. Moreover, some of the selected probiotic candidate genera are closely associated with Symbiodiniaceae or other algae. For example *Ruegeria pomeroyi* forms a symbiosis with the diatom *Thalassosira pseudonana*, providing it with the essential vitamin B_12_ [116]. Symbiodiniaceae and corals cannot generate vitamin B_12_ [117], but require it as a cofactor for enzyme functioning in central metabolism [118]. For instance, cultured Symbiodiniaceae can grow without vitamin B_12_ addition to the culture medium so long as bacteria are present [119]. Thus, the proposed probiotic candidate *Ruegeria* sp. DMG2200320 might also contribute to coral holobiont functioning by providing the essential vitamin B_12_ to Symbiodiniaceae and the coral. The genera *Muricauda, Roseibium* and *Roseovarius* sp. are associated with different Symbiodiniaceae species in culture [120]. Using probiotic candidates which are co-localised with Symbiodiniaceae is particularly appealing as excess production of ROS and RNS mostly occurs there. In a recent study, *Roseovarius* sp. with high ROS-scavenging ability isolated from Symbiodiniaceae contributed to Symbiodiniaceae growth under elevated temperatures after inoculation (Heric K, Maire J, Deore P, Perez-Gonzalez A, van Oppen MJH: Inoculation with Roseovarius increases thermal tolerance of the coral photosymbiont, Breviolum minutum, under review), further suggesting that strains of this genus could be beneficial for coral probiotics.

## Conclusions

The current study adds to the culture collection and publicly available genomes of coral-associated bacterial strains. Pure cultures are crucial for probiotic inoculation experiments [121], and bacterial genome sequences provide insights into bacterial functional potential and the relevance of bacteria to the coral holobiont. Since *G. fascicularis* has gained increased attention as an emerging coral model in recent years, this collection will support research aimed at establishing this model.

We focused on bacteria with coral bleaching mitigation features via ROS and RNS-scavenging and provide an in-depth list of putative beneficial functions of bacteria isolated from *G. fascicularis*, some of which are novel for certain bacterial genera. We provide novel insights into the potential of coral-associated bacteria to export carbon. The functionality of each trait, as well as the impacts of proposed probiotic strains on coral holobiont performance during thermal stress remains to be assessed in controlled inoculation experiments. Temporal stability and localisation of the probiotic candidates within the coral holobiont also remains to be investigated. While the field of coral probiotics is still in its infancy and functioning of bacteria within the coral holobiont is not well understood, this study provides an important step for identifying suitable probiotic bacterial strains aimed at building coral climate resilience.

### Supplementary Information

**Additional file 1. **

**Additional file 2. **

**Additional file 3. **

## Data Availability

The dataset analysed during the current study is available in the NCBI repository, under the BioProject ID PRJNA940323 (https://www.ncbi.nlm.nih.gov/bioproject/940323). The accession number for each assembled genome is provided in Supplementary Table 2.
