# Supplementary material for: Genomic exploration of coral-associated bacteria: identifying probiotic candidates to increase coral bleaching resilience in Galaxea fascicularis
Source: Microbiome. 2023 Aug 19;11:185. doi: 10.1186/s40168-023-01622-x (PMC10439622; doi:10.1186/s40168-023-01622-x)
Supplement: Supplementary file 2 — Additional file 1. [file 40168_2023_1622_MOESM1_ESM.docx]

# Supplementary Material:

# Genomic exploration of coral-associated bacteria: identifying probiotic candidates to increase coral bleaching resilience in *Galaxea fascicularis*

Authors and Affiliations:

Talisa Doering^1*^, Kshitij Tandon^1^, Sanjida H Topa^1^, Sacha J Pidot^2^, Linda L Blackall^1^ and Madeleine JH van Oppen^1,3^

^1^School of BioSciences, The University of Melbourne, Parkville, Victoria, Australia.

^2^Department of Microbiology and Immunology at the Peter Doherty Institute for Infection and Immunity, The University of Melbourne, Parkville, Victoria, Australia.

^3^Australian Institute of Marine Science, Townsville, Queensland, Australia.

*Correspondence:

Talisa Doering

tdoring@student.unimelb.edu.au

Mobile: +61 491190045

ORCID: 0009-0002-0210-8755

## Supplementary results

#### Complete metabolic pathways identified in G. fascicularis-associated bacteria

Metabolic pathways (KEGG pathway modules) that were found to be ≥80% complete in one or more bacterial isolates included amino acid metabolism, biosynthesis of terpenoids and polyketides, carbohydrate metabolism, energy metabolism, glycan metabolism, lipid metabolism, metabolism of cofactors and vitamins, nucleotide metabolism (Supplementary Figure 2). Pathways involved in metabolism of amino acids, such as arginine and proline metabolism, were found to be 50-100% complete across all bacterial families, except for Flavobacteriaceae. In turn, all eight isolates of Flavobacteriaceae exclusively showed 100% completeness in cysteine biosynthesis (M00338, KEGG pathway module). Regarding other pathways involved in amino acid metabolism, all nine isolates of Bacillaceae showed 100% completeness of methionine biosynthesis (M00017; including *Photobacterium aquae* (Vibrionaceae) and *Pseudoalteromonas lipolytica* (Alteromonadaceae)), methionine salvage pathway (M00034; including *Paenibacillus* sp. (Paenibacillaeae)), cysteine biosynthesis from methionine (M00609), lysine biosynthesis (M00525) and polyamine biosynthesis (M00133; including in *Paenibacillus* sp. (Paenibacillaeae) and *Pseudoalteromonas lipolytica* (Alteromonadaceae)). Another set of six isolates showed 100% complete pathways of both phenylalanine (M00024) and tyrosine (M00025) biosynthesis (*Pseudomonas* sp. (Pseudomonadaceae), three isolates of Vibrionaceae (*Vibrio alginolyticus*), two isolates of Alteromonadaceae (*Alteromonas* sp.). The gamma-aminobutyrate shunt was found to be 100% complete in only two isolates, *Roseibium* sp. (Stappiaceaceae) and *Microbacterium oxydans* (Microbacteriaceae). The biosynthesis of betaine (M0555) was 100% complete in *Nocardioides* sp. (Nocardioidaceae), *Marihabitans melonis* (Dermatophilaceae), in eight isolates of Bacillaceae (*Bacillus pumilus*, all seven *Bacillus safensis* isolates), both Oleiphilaceae isolates (*Marinobacter salarius, Marinobacter salsuginis*), eight isolates of Vibrionaceae (*Vibrio* sp., *Vibrio alginolyticus*, *Vibrio brasiliensis*, *Vibrio harveyi*, *Vibrio rotiferanus*), nine isolates of Alteromonadaceae (*Pseudoalteromonas lipolytica*, *Alteromonas macleodii*), all three isolates of Stappiaceae (*Roseibium* sp., two isolates of *Pseudovibrio denitrificans*) and eight isolates of Rhodobacteraceae (*Leisingera* sp., *Shimia* sp., *Roseovarius* sp., JABSSA01, two *Ruegeria* sp. isolates, two isolates of *Ruegeria arenilitoris*). In all these mentioned Bacillaceae, Oleiphilaceae, Stappiaceae and Vibrionaceae isolates, we also identified transport systems for glycine betaine (M00208; Supplementary Table 3).

A pathway involved in the biosynthesis of the secondary metabolite bacilysin (M00787) was found to be complete in all isolates of Bacillaceae only. Among metabolic pathways related to biosynthesis of terpenoids and polyketides, avermectin biosynthesis (M00777) was solely 100% complete in four isolates (*Nocardioides* sp. (Nocardioidaceae) and three isolates of Rhodobacteraceae (*Roseovarius* sp., two isolates of *Ruegeria* sp.)). Further, 100% completeness of the biosynthesis of C10-C20 isoprenoid (M00365) was found exclusively in five isolates comprising two isolates of Flavobacteriaceae (*Euzebyella* sp., *Tenacibaculum* sp.), *Microbacterium oxydans* (Microbacteriaceae), *Nocardioides* sp. (Nocardioidaceae) and *Marihabitans melonis* (Dermatophilaceae).

The distribution of pathways related to carbohydrate metabolism followed a different trend - while the majority of all bacterial isolates featured 80-100% complete pathways involved in the metabolism of central carbohydrates via, e.g. glycolysis (M00001, M0002) or gluconeogenesis (M0003), pathways related to metabolism of other, non-central carbohydrates were only complete in a few groups of isolates. For instance, a set of isolates showed 100% complete pathways of both nucleotide sugar biosynthesis (M00554) and galactose degradation (M00632). This group contained two isolates of Flavobacteriaceae (*Tenacibaculum* sp.), *Microbacterium oxydans* (Microbacteriaceae), six isolates of Vibrionaceae (*Photobacterium aquae*, *Vibrio* sp., *Vibrio brasiliensis*, *Vibrio harveyi*, *Vibrio rotiferianus*), ten isolates of Alteromonadaceae (*Alteromonas* sp*., Alteromonas macleodii*), two isolates of Sphingomonadaceae (*Qipengyuania* sp.). Furthermore, 24 isolates featured 100% complete biosynthesis of glycogen (M00854; *Winogradskyella* sp. (Flavobacteriaceae), *Paenibacillus* sp. (Paenibacillaeae), *Priestia flexa* (Bacillaceae), all nine isolates of Vibrionaceae, all 11 isolates of Alteromonadaceae and *Roseivivax marinus* (Rhodobacteraceae)). Of these isolates, 17 also exhibited a complete pathway of glycogen degradation (M00855), comprising all nine isolates of Vibrionaceae, six isolates of Alteromonadaceae and *Roseivivax marinus* (Rhodobacteraceae).

Pathways that were linked to energy metabolism such as carbon metabolism, nitrogen metabolism, photosynthesis, or sulfur metabolism were found to be complete in a few groups of isolates. Regarding carbon metabolism, the crassulacean acid metabolism (CAM), also known as CAM photosynthesis, both for dark (M00168) and light (M00169) conditions were found to be 100% complete in *Muricauda* sp. (Flavobacteriaceae), *Paenibacillus* sp. (Paenibacillaeae), all five isolates of Sphingomonadaceae (*Qipengyuania* sp.), two isolates of Rhodobacteraceae (*Ruegeria* sp., *Leisingera* sp.). CAM photosynthesis for light conditions only was additionally found to be complete in *Terasakiella* sp. (Teraskiellaceae), all three isolates of Stappiaceae and all Rhodobacteraceae (26 isolates). As CAM photosynthesis is not known in bacteria but in plants only, we suggest that both light and dark CAM photosynthesis are not functional in the identified bacterial strains. However, the individual genes that were assigned to these two pathways might be fully functional, since, for instance, phosphoenolpyruvate carboxylase (EC:4.1.1.31) is known in bacteria and converts phosphoenolpyruvate and bicarbonate into oxaloacetate and inorganic phosphorus [1]. Another pathway involved in carbon metabolism, the phosphate acetyltransferase-acetate kinase pathway (M00579), was 100% complete in 55% of all bacterial isolates, such as *Microbacterium oxydans* (Microbacteriaceae), *Psychrobacter* sp. (Moraxellaceae), *Pseudomonas* sp. (Pseudomonadaceae), *Marinobacter salsuginis* (Oleiphilaceae), all isolates of Bacillaceae and Vibrionaceae, and eight isolates of Rhodobacteraceae (*Ruegeria* sp., *Ruegeria arenilitoris*, *Ruegeria atlantica*). Pathways involved in nitrogen metabolism, such as denitrification (M00529), was 100% complete in ten isolates of Rhodobacteraceae (*Ruegeria* sp., *Ruegeria arenilitoris*, *Ruegeria atlantica*, *Aliiroseovarius* sp., *Leisingera* sp., *Cribrihabitans* sp., *Shimia* sp.) in two isolates of Stappiaceae (*Pseudovibrio denitrificans*) and in *Terasakiella* sp. (Terasakiellaceae). A 100% completeness of dissimilatory nitrate reduction (M00530) was also exhibited in ten isolates of Rhodobacteraceae (*Ruegeria* sp., *Ruegeria arenilitoris*, *Ruegeria atlantica*, *Cribrihabitans* sp., *Shimia* sp., *Thalassobius autumnalis*), *Roseibium* sp. (Stappiaceae), *Tenacibaculum* sp. (Flavobacteriaceae) and all nine isolates of Vibrionaceae. Only four isolates contained an anoxygenic photosystem II (M00597), i.e. *Roseibium* sp. (Stappiaceae) and three isolates of Rhodobacteraceae (*Roseivivax marinus*, *Roseovarius* sp.). With reference to sulfur metabolism, two pathways were found to be 100% complete in multiple bacterial isolates. First, assimilatory sulfate reduction (M00176) was complete in in *Paenibacillus* sp. (Paenibacillaeae) and in all isolates of Bacillaceae, Vibrionaceae and Alteromonadaceae. Second, thiosulfate oxidation (M00595) was 100% complete in 13 isolates of Rhodobacteraceae (e.g., *Aliiroseovarius* sp., *Leisingera* sp., *Phaeobacter italicus*, *Roseivivax marinus*, *Roseovarius* sp., *Ruegeria* sp., *Shimia* sp., *Sulfitobacter* sp., *Thalassobius autumnalis*) and in all three isolates of Stappiaceae.

Seventeen pathways involved in the metabolism of cofactors and vitamins were found to be 100% complete across one or more *G. fascicularis*-associated bacterial isolates. Among those, the biosynthesis of cobalamin (vitamin B12; M00122) was 100% complete in *Tenacibaculum* sp. (Flavobacteriaceae), *Marihabitans melonis* (Dermatophilaceae), *Paenibacillus* sp. (Paenibacillaeae), *Alcanivorax profundi* (Alcanivoracaceae), *Marinobacter salaries* (Oleiphilaceae), six isolates of Vibrionaceae (*Photobacterium aquae, Vibrio alginolyticus, Vibrio harveyi*), *Pseudoalteromonas lipolytica* (Alteromonadaceae), *Terasakiella* sp. (Teraskiellaceae), and three isolates of Rhodobacteraceae (*Ruegeria* sp., *Shimia* sp.).

Transport systems were also limited to a subset of strains. Vitamin B12 transport systems were exclusively found in all isolates of Vibrionaceae (Supplementary Table 3). One putative ATP binding cassette (ABC) transport system (M00211) was solely detected in *Roseibium* sp. (Stappiaceaceae) and *Paenibacillus* sp. (Paenibacillaeae), while another putative ABC transport system (M00247) was identified in seven isolates of Vibrionaceae (*Vibrio* sp., *Vibrio alginolyticus*, *Vibrio rotiferianus*, *Vibrio harveyi*), two isolates of Oleiphilaceae (*Marinobacter salarius*, *Marinobacter salsuginis*), *Teraskiella* sp. (Terasakiellaceae), *Psychrobacter* sp. (Moraxellaceae) and again in *Paenibacillus* sp. (Paenibacillaeae; Supplementary Figure 2).

## Supplementary Tables

**Supplementary Table 1** Cultured bacterial isolates from Great Barrier Reef-sourced G. fascicularis.

| Genus | Class | No. of cultures | No. of species | Species (closest hit in NBCI database via BLASTn) based on ~1000 positions of the 16S rRNA gene |
| --- | --- | --- | --- | --- |
| *Bacillus* | Bacilli | 180 | 4 | *Bacillus australimaris, Bacillus flexus, Bacillus pumilus, Bacillus safensis* |
| *Alteromonas* | Gammaproteobacteria | 153 | 8 | *Alteromonas litorea, Alteromonas macleodii, Alteromonas marina, Alteromonas mediterranea, Alteromonas australica, Alteromonas oceani, Alteromonas simiduii, Altermonas tagae* |
| *Vibrio* | Gammaproteobacteria | 70 | 10 | *Vibrio alginolyticus, Vibrio atypicus, Vibrio hepatarius, Vibrio natriegens, Vibrio brasiliensis, Vibrio harveyi, Vibrio hyugaensis, Vibrio rotiferianus, Vibrio jasicida, Vibrio mytili* |
| *Ruegeria* | Alphaproteobacteria | 50 | 5 | *Ruegeria arenilitoris, Ruegeria atlantica, Ruegeria conchae, Ruegeria lacuscaerulensis, Ruegeria profundi* |
| *Balneola* | Balneolia | 17 | 1 | *Balneola alkaliphila* |
| *Pseudovibrio* | Alphaproteobacteria | 16 | 1 | *Pseudovibrio denitrificans* |
| *Micrococcus* | Actinomycetia | 12 | 2 | *Micrococcus aloeverae, Micrococcus yunnanensis* |
| *Nonlabens* | Flavobacteriia | 12 | 2 | *Nonlabens sediminis, Nonlabens tegetincola* |
| *Erythrobacter* | Alphaproteobacteria | 8 | 3 | *Erythrobacter aquimaris, Erythrobacter flavus, Erythrobacter nanhaisediminis* |
| *Sphingomonas* | Alphaproteobacteria | 8 | 2 | *Sphingomonas dokdonensis, Sphingomonas sanguinis* |
| *Thalassobius* | Alphaproteobacteria | 8 | 1 | *Thalassobius mediterraneus* |
| *Phaeobacter* | Alphaproteobacteria | 7 | 2 | *Phaeobacter italicus, Phaeobacter piscinae* |
| *Euzebyella* | Flavobacteriia | 5 | 1 | *Euzebyella saccharophila* |
| *Shimia* | Alphaproteobacteria | 5 | 1 | *Shimia marina* |
| *Staphylococcus* | Bacilli | 4 | 3 | *Staphylococcus pasteuri, Staphylococcus warneri, Staphylococcus epidermis* |
| *Tenacibaculum* | Flavobacteriia | 4 | 2 | *Tenacibaculum aiptasiae, Tenacibaculum litopenaei* |
| *Brachybacterium* | Actinomycetia | 3 | 2 | *Brachybacterium saurashtrense, Brachybacterium paraconglomeratum* |
| *Kocuria* | Actinomycetia | 3 | 2 | *Kocuria palustris, Kocuria rhizophila* |
| *Marinobacter* | Gammaproteobacteria | 3 | 2 | *Marinobacter algicola, Marinobacter salsuginis* |
| *Microbacterium* | Actinobacteria | 3 | 1 | *Microbacterium saperdae* |
| *Roseovarius* | Alphaproteobacteria | 3 | 2 | *Roseovarius aestuarii, Roseovarius indicus* |
| *Alcanivorax* | Gammaproteobacteria | 2 | 1 | *Alcanivorax nanhaiticus* |
| *Epibacterium* | Alphaproteobacteria | 2 | 2 | *Epibacterium multivorans, Epibacterium ulvae* |
| *Loktanella* | Alphaproteobacteria | 2 | 1 | *Loktanella acticola* |
| *Muricauda* | Flavobacteriia | 2 | 1 | *Muricauda aquimarina* |
| *Pseudoalteromonas* | Gammaproteobacteria | 2 | 1 | *Pseudoalteromonas shioyasakiensis* |
| *Winogradskyella* | Flavobacteriia | 2 | 1 | *Winogradskyella poriferorum* |
| *Aliiroseovarius* | Alphaproteobacteria | 1 | 1 | *Aliiroseovarius zhejiangensis* |
| *Amphritea* | Gammaproteobacteria | 1 | 1 | *Amphritea spongicola* |
| *Amylibacter* | Alphaproteobacteria | 1 | 1 | *Amylibacter lutimaris* |
| *Aquimarina* | Flavobacteria | 1 | 1 | *Aquimarina penaei* |
| *Arthrobacter* | Actinomycetia | 1 | 1 | *Arthrobacter luteolus* |
| *Blastomonas* | Alphaproteobacteria | 1 | 1 | *Blastomonas marina* |
| *Brevundimonas* | Alphaproteobacteria | 1 | 1 | *Brevundimonas vesicularis* |
| *Cribrihabitans* | Alphaproteobacteria | 1 | 1 | *Cribrihabitans marinus* |
| *Dermacoccus* | Actinomycetia | 1 | 1 | *Dermacoccus profundi* |
| *Janibacter* | Actinobacteria | 1 | 1 | *Janibacter melonis* |
| *Labrenzia* | Alphaproteobacteria | 1 | 1 | *Labrenzia alba* |
| *Leisingera* | Alphaproteobacteria | 1 | 1 | *Leisingera aquimarina* |
| *Marimonas* | Alphaproteobacteria | 1 | 1 | *Marimonas arenosa* |
| *Marivita* | Alphaproteobacteria | 1 | 1 | *Marivita litorea* |
| *Microbulbifer* | Gammaproteobacteria | 1 | 1 | *Microbulbifer agarilyticus* |
| *Nocardioides* | Actinomycetia | 1 | 1 | *Nocardioides cavernae* |
| *Paenibacillus* | Bacilli | 1 | 1 | *Paenibacillus pabuli* |
| *Paracoccus* | Alphaproteobacteria | 1 | 1 | *Paracoccus acridae* |
| *Photobacterium* | Gammaproteobacteria | 1 | 1 | *Photobacterium aquae* |
| *Pseudomonas* | Gammaproteobacteria | 1 | 1 | *Pseudomonas pachastrellae* |
| *Pseudotenacibaculum* | Flavobacteriia | 1 | 1 | *Pseudotenacibaculum haliotis* |
| *Psychrobacter* | Gammaproteobacteria | 1 | 1 | *Psychrobacter submarinus* |
| *Roseivivax* | Alphaproteobacteria | 1 | 1 | *Roseivivax marinus* |
| *Roseobacter* | Alphaproteobacteria | 1 | 1 | *Roseobacter ponti* |
| *Sulfitobacter* | Alphaproteobacteria | 1 | 1 | *Sulfitobacter noctilucicola* |
| *Terasakiella* | Alphaproteobacteria | 1 | 1 | *Terasakiella brassicae* |
| *Tropicibacter* | Alphaproteobacteria | 1 | 1 | *Tropicibacter naphthalenivorans* |
| TOTAL |  | **613** | **91** |  |

**Supplementary Table 2** Characteristics of assembled bacterial genomes of G. fascicularis. (Excel Sheet)

**Supplementary Table 3** Metabolic pathways that were >80% complete in one or more bacterial genomes of G. fascicularis. Pathways were annotated with MetabolicG and completeness was estimated using EnrichM. (Excel Sheet)

## Supplementary Figures


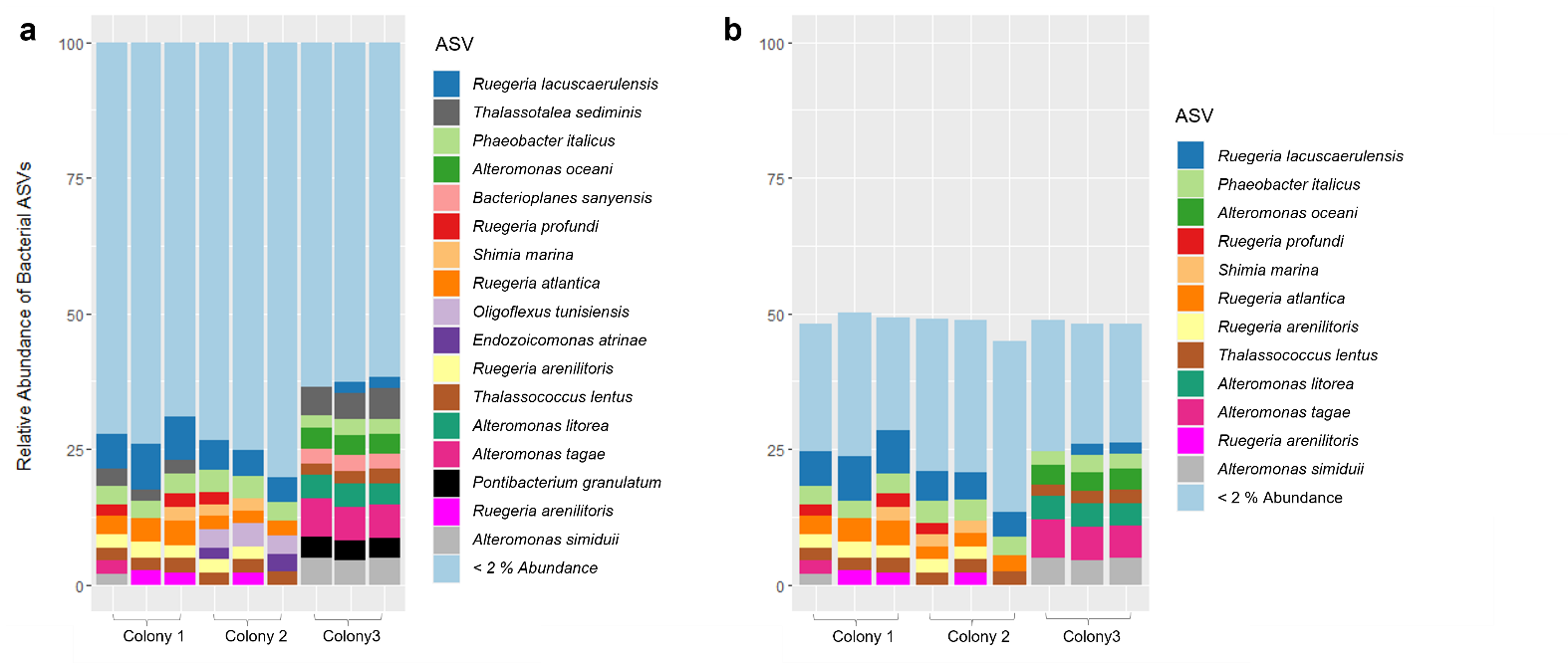


**Supplementary Figure 1** Bacterial community composition of G. fascicularis colonies that were used for culturing (16S rRNA gene metabarcoding dataset). **A)** Amplicon sequence variants (ASVs) of G. fascicularis colonies that were used for bacterial culturing and **B)** the fraction of bacterial ASVs of G. fascicularis colonies whose bacterial genus was obtained in pure culture. Compositions are shown per sample per G. fascicularis colony (n=3 per colony). ASVs are listed as their closest hit in the NBCI database via BLASTn. Less abundant bacteria are grouped in one category (< 2 % relative abundance, light blue).


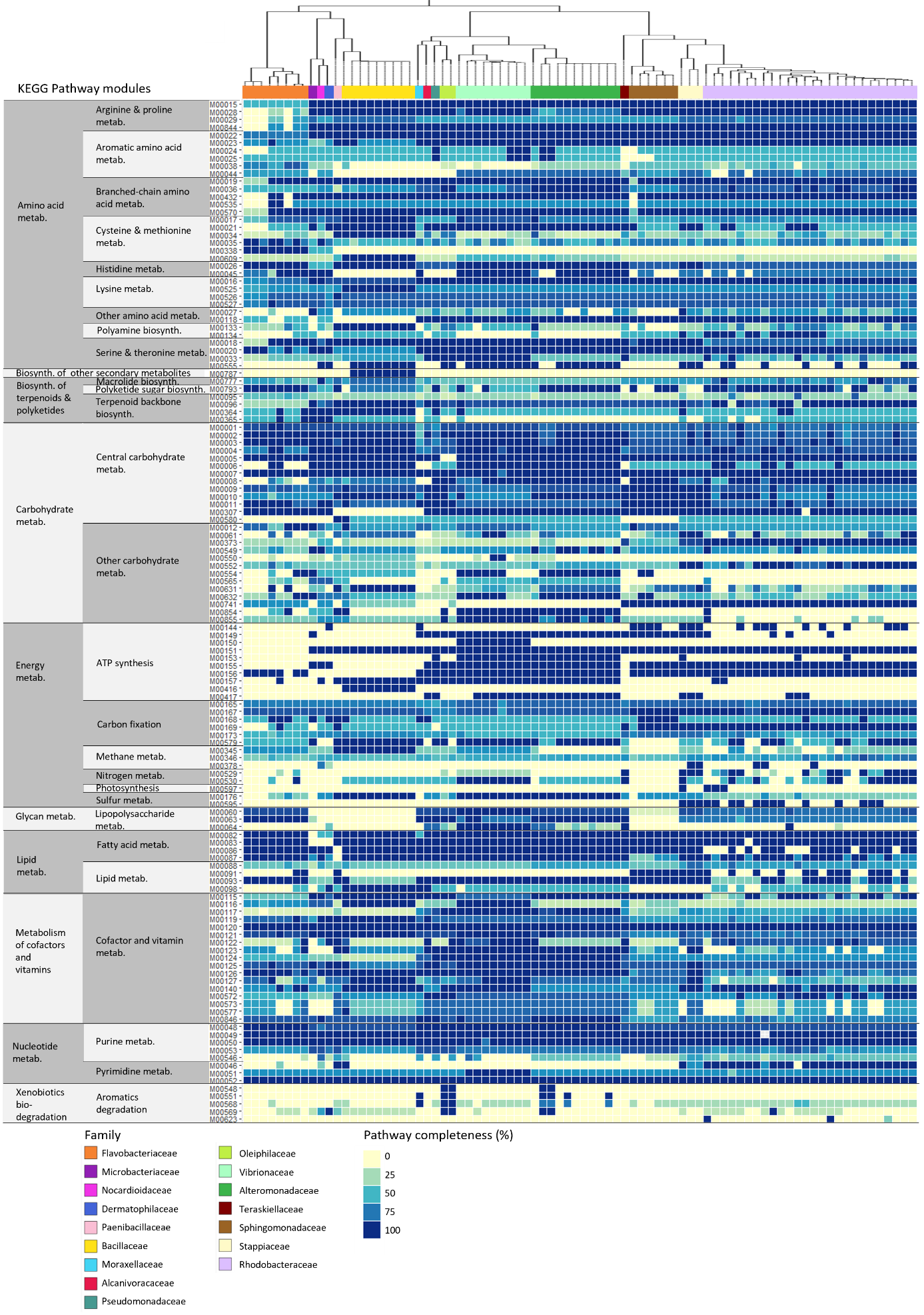


**Supplementary Figure 2** Metabolic pathways (KEGG module database) found to be ≥80% complete in one or more G. fascicularis-associated bacterial genomes. Pathways were annotated using MetabolicG and pathway completeness (in %) was estimated in EnrichM. Metab. = metabolism, biosynth. = biosynthesis.


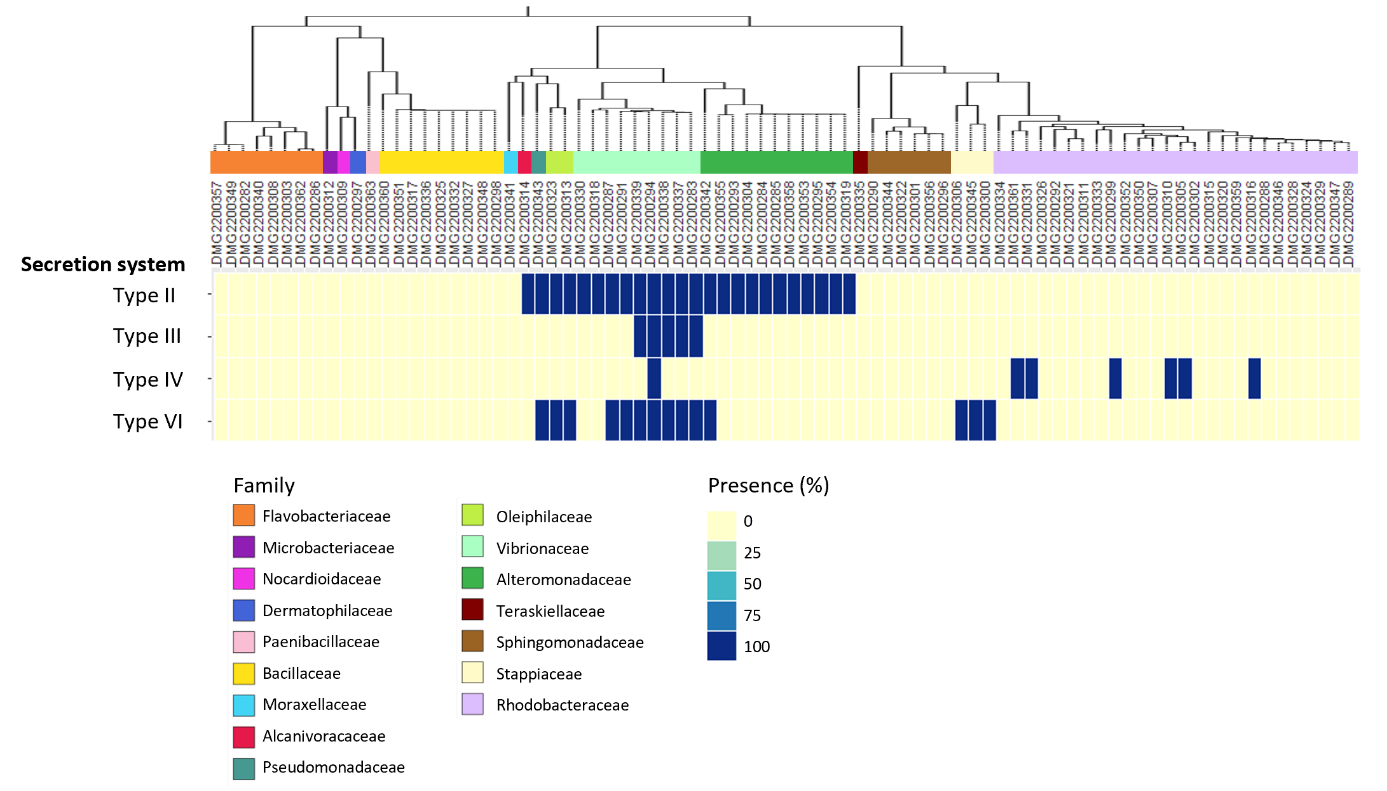


**Supplementary Figure 3** Secretion systems found in G. fascicularis-associated bacteria. Secretion systems were annotated using MetabolicG and completion (in %) of each secretion system was estimated using EnrichM.


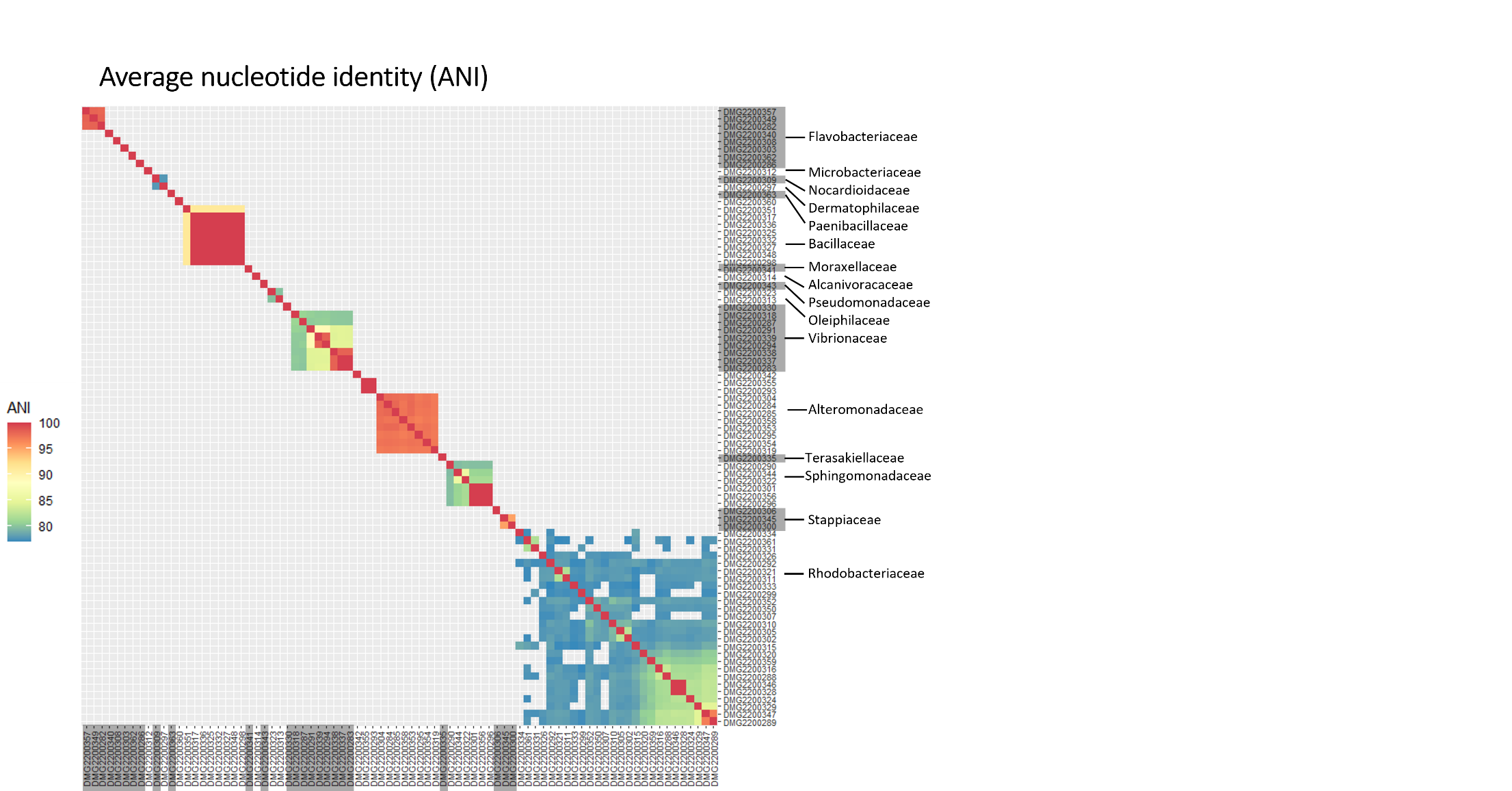


**Supplementary Figure 4** Average nucleotide identity between all 82 bacterial genomes from G. fascicularis, visualized per bacterial family.


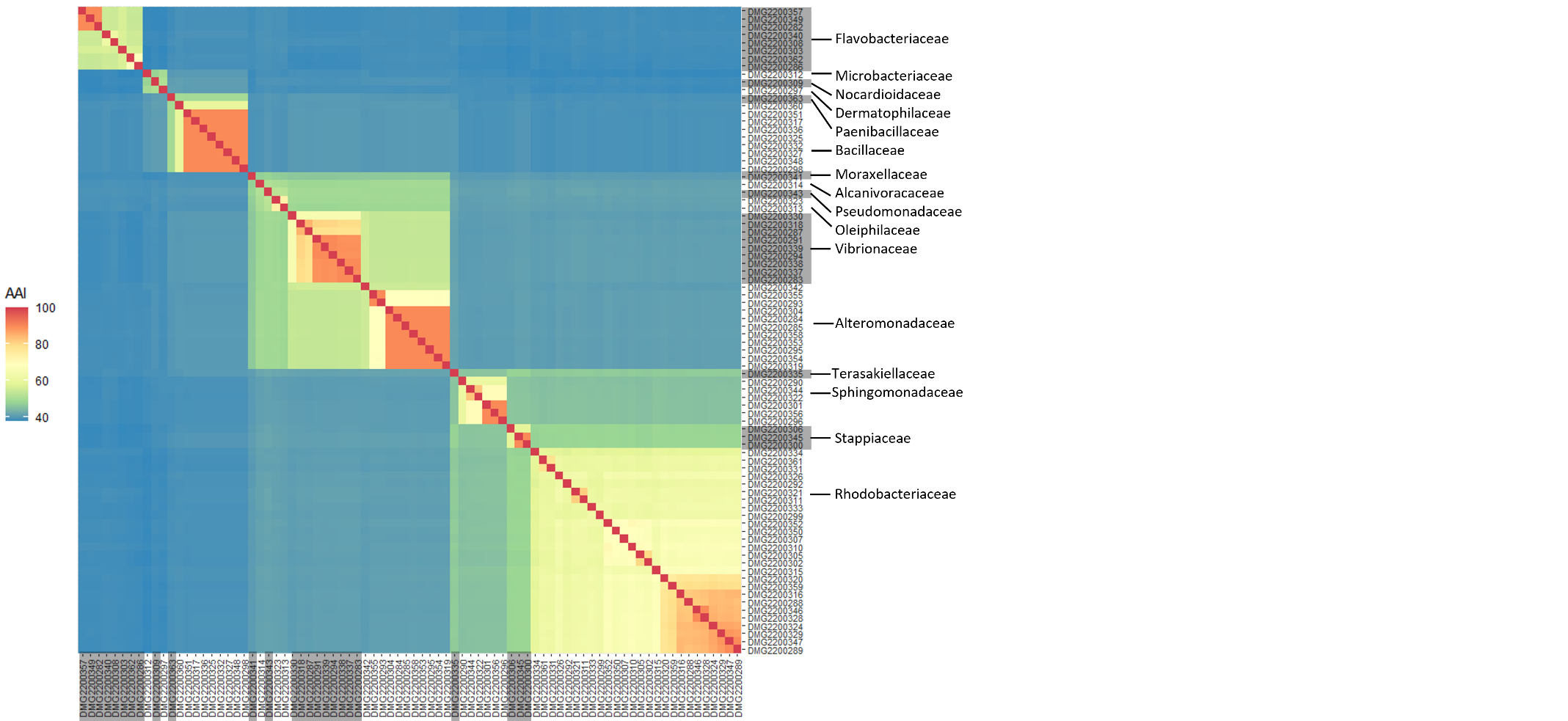


**Supplementary Figure 5**  Amino acid identity between all 82 bacterial genomes from G. fascicularis, visualized per bacterial family.


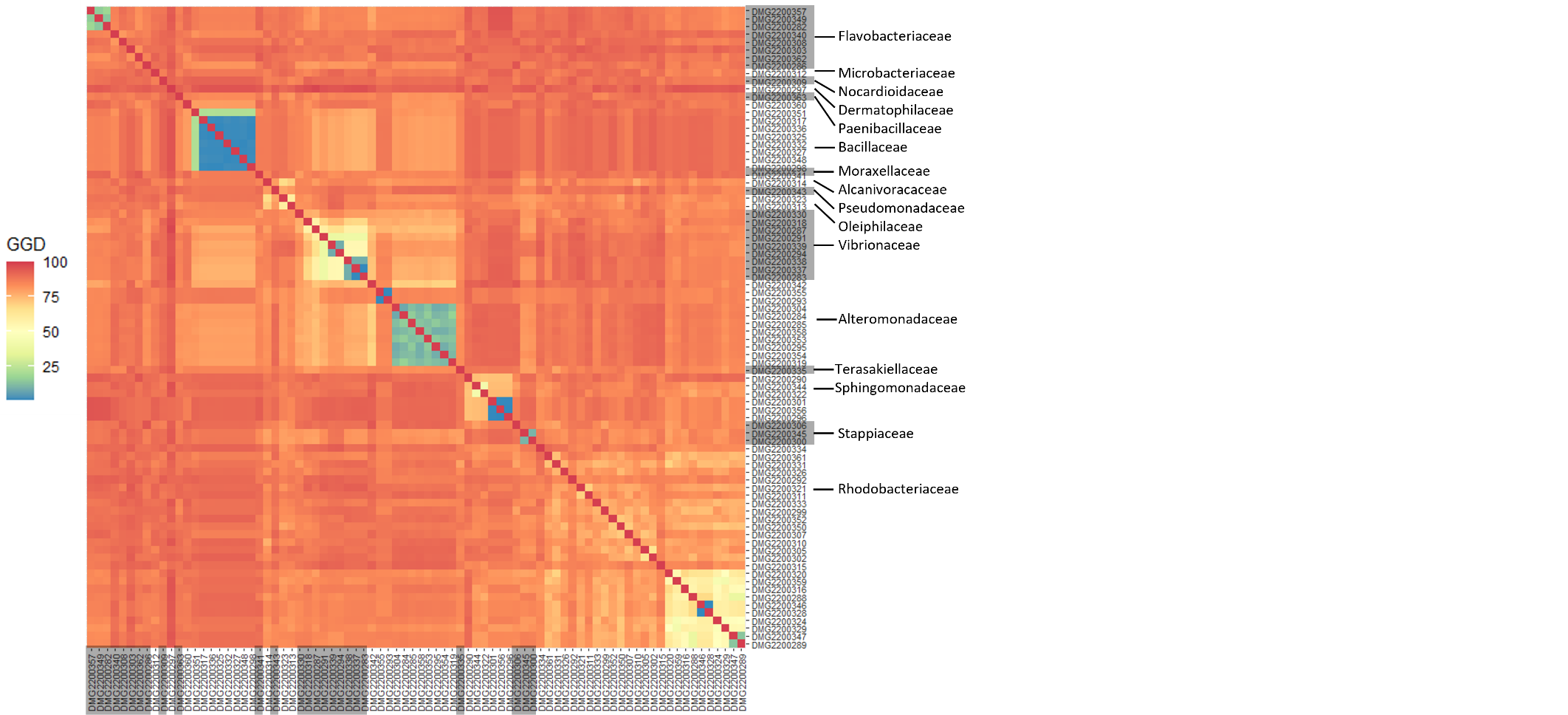


**Supplementary Figure 6**  Genome-genome distance between all 82 bacterial genomes from G. fascicularis, visualized per bacterial family.

## References

1. Inui M, Dumay V, Zahn K, Yamagata H, Yukawa H. Structural and functional analysis of the phosphoenolpyruvate carboxylase gene from the purple nonsulfur bacterium *Rhodopseudomonas palustris* No. 7. J Bacteriol. 1997;179:4942–5.
